# Cognitive safety of focused ultrasound thalamotomy for tremor: 1-year follow-up results of the COGNIFUS part 2 study

**DOI:** 10.3389/fneur.2024.1395282

**Published:** 2024-06-17

**Authors:** Gennaro Saporito, Patrizia Sucapane, Federico Bruno, Alessia Catalucci, Carlo Masciocchi, Maria Letizia Pistoia, Alessandra Splendiani, Alessandro Ricci, Ernesto Di Cesare, Carmine Marini, Monica Mazza, Rocco Totaro, Francesca Pistoia

**Affiliations:** ^1^Department of Biotechnological and Applied Clinical Sciences, University of L’Aquila, L’Aquila, Italy; ^2^Department of Neurology, San Salvatore Hospital, L’Aquila, Italy; ^3^Department of Radiology, San Salvatore Hospital, L'Aquila, Italy; ^4^Department of Neurosurgery, San Salvatore Hospital, L’Aquila, Italy; ^5^Department of Internal Medicine, Public Health, Life and Environmental Sciences, University of L’Aquila, L’Aquila, Italy

**Keywords:** tremor, cognitive outcomes, Parkinson disease, essential tremor, MRgFUS

## Abstract

**Introduction:**

In the COGNitive in Focused UltraSound (COGNIFUS) study, we examined the 6-month cognitive outcomes of patients undergoing MRgFUS thalamotomy. This study endorsed the safety profile of the procedure in terms of cognitive functions that cannot be evaluated in real-time during the procedure unlike other aspects. The aim of the COGNIFUS Part 2 study was to investigate the cognitive trajectory of MRgFUS patients over a 1-year period, in order to confirm long-term safety and satisfaction.

**Methods:**

We prospectively evaluated the cognitive and neurobehavioral profile of patients with essential tremor (ET) or Parkinson’s Disease (PD) related tremor undergoing MRgFUS thalamotomy at 1 year-follow-up following the treatment.

**Results:**

The sample consists of 50 patients (male 76%; mean age ± SD 69.0 ± 8.56; mean disease duration ± SD 12.13 ± 12.59; ET 28, PD 22 patients). A significant improvement was detected at the 1 year-follow-up assessment in anxiety and mood feelings (Hamilton Anxiety rating scale 5.66 ± 5.02 vs. 2.69 ± 3.76, *p* ≤ <0.001; Beck depression Inventory II score 3.74 ± 3.80 vs. 1.80 ± 2.78, *p* = 0.001), memory domains (Rey Auditory Verbal Learning Test, immediate recall 31.76 ± 7.60 vs. 35.38 ± 7.72, *p* = 0.001 and delayed recall scores 5.57 ± 2 0.75 vs. 6.41 ± 2.48), frontal functions (Frontal Assessment Battery score 14.24 ± 3.04 vs. 15.16 ± 2.74) and in quality of life (Quality of life in Essential Tremor Questionnaire 35.00 ± 12.08 vs. 9.03 ± 10.64, *p* ≤ 0.001 and PD Questionnaire −8 7.86 ± 3.10 vs. 3.09 ± 2.29, *p* ≤ 0.001).

**Conclusion:**

Our study supports the long-term efficacy and cognitive safety of MRgFUS treatment for ET and PD.

## 1 Introduction

Tremor is the cardinal sign of essential tremor (ET) and one of the most disabling symptoms of Parkinson’s disease (PD). When tremor is refractory to pharmacological therapy, it may benefit from surgical approaches like radiofrequency thalamotomy, gamma knife thalamotomy, and thalamic stimulation (1). Magnetic resonance-guided focused ultrasound (MRgFUS) thalamotomy is a more recent approach that combines two technologies: magnetic resonance (MR) imaging and focused ultrasound (FUS). This combination allows obtaining a precise targeting of the ventral intermediate (Vim) nucleus and subsequent Vim ablation through high-intensity ultrasound waves. To date, many studies confirmed the efficacy and safety of MRgFUS thalamotomy for the treatment of medically refractory ET and PD-related tremor (2–5). Since the thalamus also plays an important role in cognition, evaluating the patient’s cognitive dimension is considered worthy of careful assessment both in the short and long term. In this respect, some studies reported a worsening in processing speed, executive function, memory and verbal fluency following unilateral thalamotomy using various techniques (6–10). Other reported stable or even improved cognitive performances in these same domains (11, 12). Moreover, a recent metanalysis analyzed the results of eight studies in this field, including 193 patients with ET, PD, or multiple sclerosis managed with MRgFUS, Radiofrequency ablation or Gamma Knife radiosurgery (13). When considering the whole sample, regardless of the technique used, a small but significant decline in phonemic fluency and a trend toward a decline in semantic fluency were observed, while the other domains remained unchanged (13). Conversely, when restricting the analysis to studies using MRgFUS, no evidence of cognitive decline across any domain was found (13). In the *COGNitive in Focused UltraSound (COGNIFUS)* study, we later investigated the 6-month cognitive outcomes of patients undergoing MRgFUS thalamotomy, showing an improvement in anxiety feelings and in quality of life without changes in frontal and executive functions, verbal fluency and memory, and abstract reasoning and problem-solving abilities (14). The aim of the COGNIFUS Part 2 study was to investigate the cognitive trajectory of MRgFUS patients over a 1-year period, in order to confirm long-term safety and satisfaction.

## 2 Materials and methods

### 2.1 Protocol and study population

This prospective study included patients who underwent MRgFUS VIM thalamotomy for medically refractory ET and PD-related tremor within a 2-year period and receiving a complete neuropsychological and behavioral assessment at 6-month and at 1 year following the treatment. Criteria to be included in the study were: (i) age > 18 years, (ii) signed informed consent to be enrolled in the study, and (iii) availability to attend the intermediate 6-month visit and the final 1-year visit following MRgFUS thalamotomy. Exclusion criteria were a previous history of neurological or psychiatric disorders, and a history of deep brain stimulation (DBS) or previous stereotactic ablation. The study was approved by the Internal Review Board of the University of L’Aquila (n. 08/22) and performed according to the declaration of Helsinki. An informed consent to participate in the study was signed by all the included patients.

### 2.2 Procedures

A complete clinical, neurobehavioral, and neuropsychological assessment was performed in all included patients before MRgFUS thalamotomy (baseline, t0), at 6 months (t1) and 1 year after the procedure (t2). All three assessments were performed in the ON state for the PD group. Main clinical variables were recorded at baseline (24–48 h before the treatment), at 6-month (t1) and at the 1-year follow-up visit (t2). The tremor improvement was quantified by assessing changes in the Fahn-Tolosa-Marin (FTM) Clinical Rating Scale for tremor (CRST) in all patients: the FTM is a scale initially designed to assess ET, that has been later validated to assess PD tremor (15, 16). The Movement Disorder Society-Sponsored Revision of the Unified Parkinson’s Disease Rating Scale (MDS-UPDRS) part III [MDS-UPDRS-III] was also administered to patients with PD (17). The neuropsychological battery included the following tests: the Montreal Cognitive Assessment (MOCA) test, the Mini Mental State Examination (MMSE), the Frontal Assessment Battery (FAB), the Rey Auditory Verbal Learning Test (RAVLT), the Single Letter-cued (phonemic) fluency (FAS) test, the Categorical Verbal Fluency test, the Raven’s Progressive Matrices (RPM), the Hamilton Anxiety rating scale (HAM-A), the Beck Depression Inventory-II (BDI-II), the Quality of life in Essential Tremor Questionnaire (QUEST), and the Parkinson’s disease Questionnaire-8 (PDQ-8) (18–30). The MOCA test and the MMSE are cognitive screening tools with good reliability in ET and PD patients: attention and concentration, executive functions, memory, language, visuoconstructional skills, conceptual thinking, calculations, and orientation are some of the cognitive domains examined (18–20). The FAB is one of the most widely used screening tool to assess executive functions: conceptualization processes, abstract reasoning, mental flexibility, motor programming, executive control, resistance to interference, inhibitory control, and environmental autonomy are some of the cognitive skills examined (21, 22). The RAVLT investigates the person’s ability to codify, consolidate, store, and retrieve verbal information depending on the integrity of attention, concentration, and short-term memory (23). The FAS test investigates executive functions and processing speed by requiring patients to name as many words as possible starting with F, A, and S in 60 s, respectively (24) while the Categorical Verbal Fluency test explores lexical retrieval and production by requiring patients to say as many words as possible belonging to the “colors,” “animals,” and “fruits” categories in three different trials, which also last 60 s each (25). Finally, the RPM test provides a non-verbal estimate of fluid intelligence and reasoning (26). The HAM-A scale and the BDI-II were used to investigate anxiety and depressive feelings (27, 28) while the QUEST and the PDQ-8 were used to measure the perceived quality of life in ET and PD patients, respectively, (29, 30). The standardization and calibration of the neuropsychological tests used, as well as the interpretation of the results according to the reference cut-off values, were carried out in accordance with the guidelines provided by the reference standards (31). The neuropsychological assessment was conducted by a certified psychologist (GS) in accordance with testing conditions ensuring privacy, adequate illumination, and a distraction-free environment, with a duration typically lasting 30–40 min.

### 2.3 Neuroradiological assessment and high intensity focused ultrasound treatment

**Figure 1:**
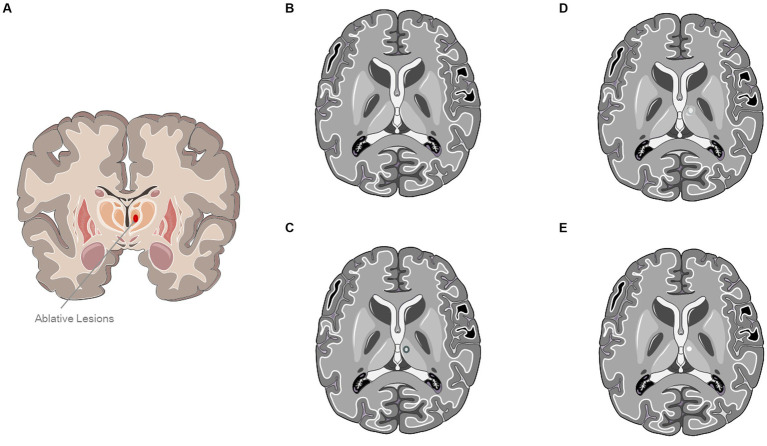
Evolution of a typical lesion on MRI at 24 h, 6 months, and 1 year after the procedure. **(A)** Ablative Lesion of Ventral Intermediate Nucleus (VIM). **(B)** A typical MRI sequence prior to ultrasound treatment. **(C)** Representation of a characteristic lesion of the VIM at 24-h after treatment. **(D)** Panel **(C)** depicts a standard MRI T2-weighted sequence obtained 6 months post-treatment. **(E)** Representation of a typical left ventral intermediate nucleus lesion 1-year post-treatment In panels **(C,D)**, a hypointense lesion characteristic of the ventral intermediate nucleus is evident. The image was partly generated using Servier Medical Art, provided by Servier, licensed under a Creative Commons Attribution 3.0 unported license.

All patients were subjected to brain CT and MRI before MRgFUS treatment to evaluate the eligibility to the procedure based on neuroimaging findings and skull density ratio (SDR) computation. The whole HIFU procedure is described in a previous publication (14). Figure 1 graphically displays the evolution of a typical lesion on MRI at 24 h, 6 months, and 1 year after the procedure.

## 3 Statistical analysis

To compare preprocedural and postprocedural scores, either a paired *t*-test or Wilcoxon signed-rank test was employed based on the normal distribution status. Pearson and Spearman correlation coefficients were calculated to examine associations between motor tests and neuropsychological or neurobehavioral tests. Repeated measures ANOVA was utilized to analyze data within the same subjects. Continuous variables were expressed as the mean ± standard deviation (SD), while categorical variables were presented as frequency or percentage. Results were deemed significant if they surpassed an alpha level of 0.003, which was adjusted according to the Bonferroni correction for the number of tests (0.05/14). Statistical analyses were conducted using JAMOVI 2.2.24 software.

## 4 Results

One hundred patients were screened for the inclusion in the study. Out of them, 50 patients were excluded as unavailable to attend the 1-year follow-up assessment. The 50% drop-out rate was mainly due to the geographic distance of patients from the location where the procedure was performed, resulting in difficulty returning 1 year later for clinical follow-up. Overall, 50 patients (males 76%; mean age ± SD 69.0 ± 8.56 years; mean disease duration ± SD 12.13 ± 12.59 years; mean education ± SD 9.58 ± 3.9 years) completed the clinical, neurobehavioral, and neuropsychological assessment at baseline, at 6-month and at the 1-year follow-up visit. The final sample was different from that reported in our previous study, making this study not a strict follow-up continuation of the previous one (14). The main clinical indication to perform thalamotomy under MRgFUS guidance was ET (*n* = 28; mean age ± SD 69.04 ± 8.0 years, mean disease duration 15.41 ± 15.0 years, mean education 9.43 ± 3–95 years) and PD-related tremor (*n* = 22; mean age ± SD 68.95 ± 9.42 years, mean disease 7.90 ± 6.85 years, mean education 9.77 ± 3.91 years). A left VIM thalamotomy was performed in 43 patients and a right VIM thalamotomy in the remainder. For the majority of patients (*n* = 45; 90%), the treated hemisphere was also the dominant one.

### 4.1 Tremor improvement

**Figure 2:**
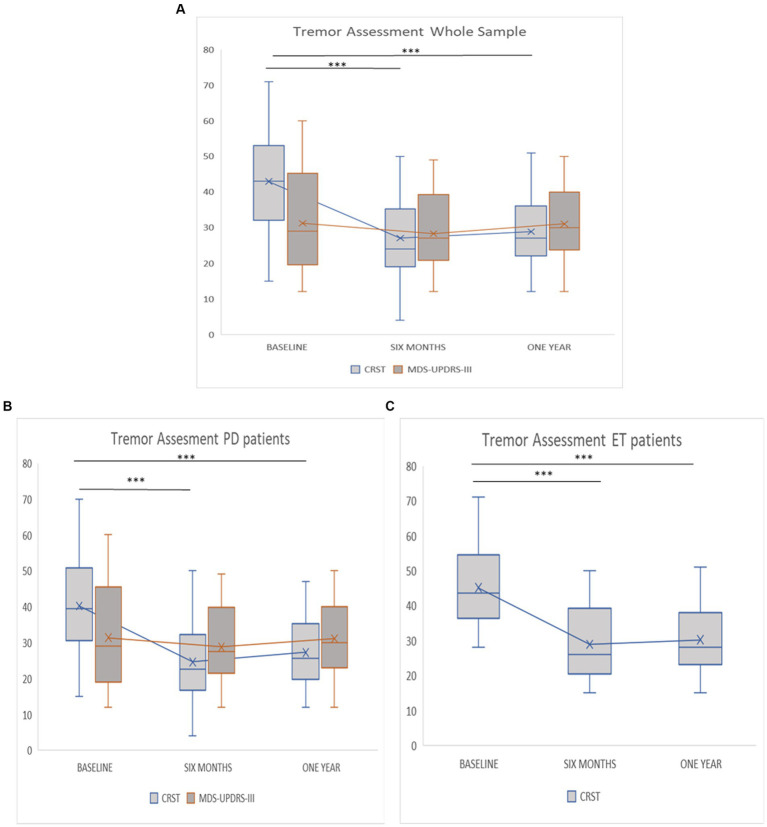
**(A–C)** Evaluation about tremor assessment at baseline, 6 months and 1 year follow-up. **(A)** Tremor assessment whole sample. **(B)** Tremor assessment PD patients. **(C)** Tremor assessment ET patients. Asterisks indicate *post-hoc* comparison (^***^ < 0.001).

When considering the entire sample without differentiating by subgroups, an improvement of the CRST total score was observed at 6 months (42.94 ± 13.67 to 27.02 ± 11.41; *Post-hoc*, *p* < 0.001) as well at 1 year (from 42.94 ± 13.67 vs. 28.68 ± 9.85, *Post-hoc*, *p* ≤ 001) following MRgFUS (Figure 2A). Conversely, the postprocedural MDS-UPDRS-III total score did not show a significant improvement at 6 months (from 31.23 ± 13.50 to 28.71 ± 10.40; *post-hoc*, *p* = 0.577) and at 1 year (from 31.23 ± 13.50 to 30.90 ± 9.46; *post-hoc p* = 1.000) following the treatment. When stratifying the whole sample by clinical diagnosis, the *post-hoc* comparisons (Figures 2B,C) indicated a significant improvement in total CRST score among patients with PD (*p* < 0.001) and ET (*p* < 0.001) at both 6 months and 1 year after treatment (Figures 2B,C).

### 4.2 Cognitive and behavioral changes

**Figure 3:**
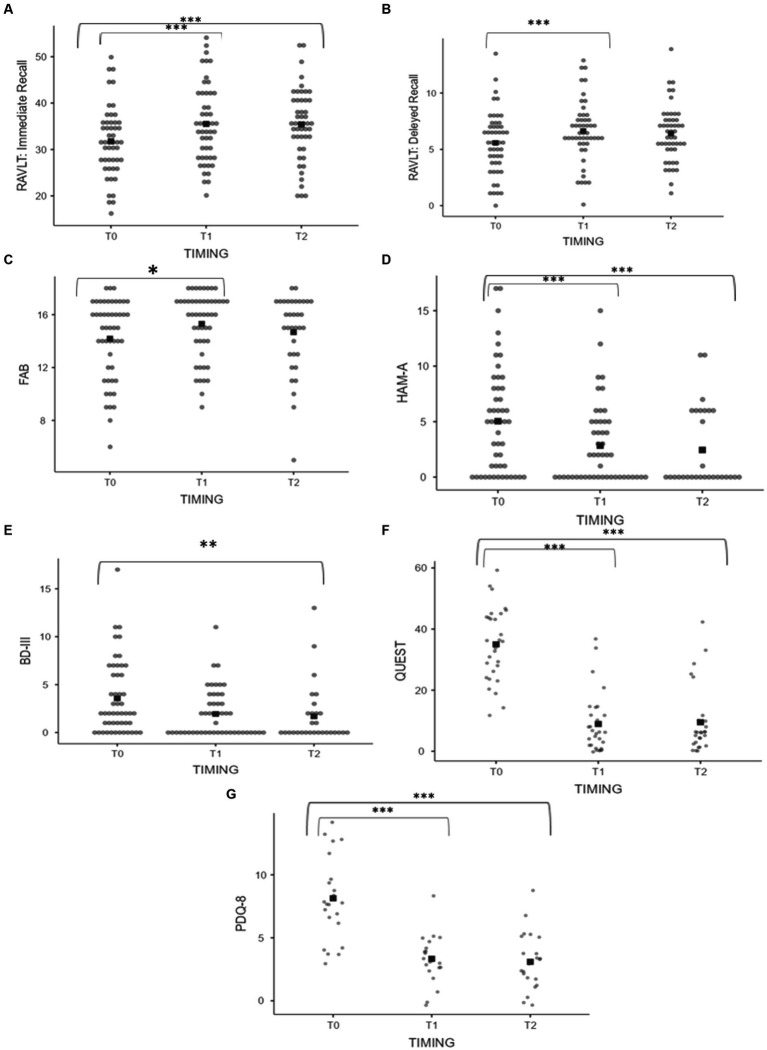
**(A–G)** Pre and postprocedural scores on neuropsychological assessment of the whole sample. **(A)** RAVLT: Immediate Recall. **(B)** RAVLT: Delayed Recall. **(C)** FAB. **(D)** HAM-A. **(E)** BDI-II. **(F)** QUEST. **(G)** PDQ-8. Asterisks indicate significant *p* value (^***^ < 0.001, ^**^0.001, and ^*^0.003).

When considering the entire sample without differentiating by subgroups, the following changes in behavioral and cognitive domains were observed at 6 months and 1 year, respectively: at 6 months, a statistically significant improvement was detected in anxiety feelings (HAM-A 5.66 ± 5.02 vs. 2.70 ± 4.09, *p* < 0.001) and in cognitive domains including memory (RAVLT: immediate recall 31.76 ± 7.60 vs. 35.51 ± 8.38; *p* ≤ 0.001; RAVLT: delayed recall 5.57 ± 2.75 vs. 7.03 ± 3.85; *p* ≤ 0.001) and frontal functions (14.24 ± 3.04 vs. 15.24 ± 2.38; *p* = 0.003). At 1 year following the treatment, an improvement was detected in anxiety and mood feelings (HAM-A 5.66 ± 5.02 vs. 2.69 ± 3.76, *p* ≤ 0.001; BDI-II 3.74 ± 3.80 vs. 1.80 ± 2.78, *p* = 0.001) and memory domains (RAVLT: Immediate recall 31.76 ± 7.60 vs. 35.38 ± 7.72, *p* = 0.001). Comparison between the mean scores is shown in Figures 3A–E. Moreover, an improvement in quality of life was detected both at 6 months (QUEST: 35.00 ± 12.08 vs. 8.93 ± 9.86, *p* ≤ 0.001; PDQ-8 7.86 ± 3.10 vs. 3.10 ± 1.52, *p* ≤ 0.001) and at 1 year (QUEST 35.00 ± 12.08 vs. 9.03 ± 10.64, *p* ≤ 0.001; PDQ-8 7.86 ± 3.10 vs. 3.09 ± 2.29, *p* ≤ 0.001) after the treatment (Figures 3F,G). Psychometric tests exploring executive functions, verbal fluency, abstract reasoning, and problem-solving abilities revealed no significant changes across multiple evaluations (Table 1).

**Table 1:** Changes in neuropsychological and neurobehavioral scores across baseline, 6-month, and 1 year follow-up for the whole sample.

Neuropsychological and neurobehavioral tests	Baseline	6-month follow-up	1 year follow-up	*p* value 6 months	*p* value 1 year
Mini Mental State Examination	27.38 ± 2.39	28.29 ± 1.70	28.33 ± 1.69	0.012	0.005
Montreal Cognitive Assessment	23.23 ± 4.93	23.78 ± 3.62	23.90 ± 3.63	0.053	0.004
Frontal Assessment Battery	14.24 ± 3.04	15.24 ± 2.38	15.16 ± 2.74	0.003	0.023
Single letter-cued (phonemic) fluency test	27.30 ± 9.76	28.32 ± 10.38	28.85 ± 9.62	0.336	0.090
Single letter-cued (semantic) fluency test	10.42 ± 2.70	10.50 ± 2.77	10.47 ± 2.85	0.774	0.908
Rey Auditory Verbal Learning Test R.I	31.76 ± 7.60	35.51 ± 8.38	35.38 ± 7.72	<001	0.001
Rey Auditory Verbal Learning Test R.D	5.57 ± 2.75	7.03 ± 3.85	6.41 ± 2.48	<001	0.011
Raven’s Progressive Matrices	28.66 ± ±4.72	28.90 ± 5.15	28.86 ± 5.15	0.460	0.686
Hamilton Anxiety rating scale	5.66 ± 5.02	2.70 ± 4.09	2.26 ± 3.76	<001	<001
Beck Depression Inventory-II	3.74 ± 3.80	1.90 ± 2.70	1.80 ± 2.78	0.006	<001

When stratifying the entire sample by subgroups, PD patients showed an improvement of anxiety feelings (HAM-A 6.14 ± 4.51 vs. 2.55 ± 2.91; *p* = 0.002) and in quality of life (PDQ-8 8.10 ± 2.97 vs. 3.11 ± 1.56; *p* ≤ 0.001) at 6-month following the procedure. The quality of life continued to show improvement at 1-year (PDQ-8 8.10 ± 2.97 vs. 3.10 ± 2.34; *p* ≤ 0.001), in combination with mood improvements (BDI-II 4.73 ± 3.30 vs. 1.68 ± 2.43; *p* = 0.003). ET patients showed an improvement of anxiety feelings (HAM-A 5.29 ± 5.44 vs. 2.50 ± 4.76; *p* = 0.001), quality of life (QUEST 34.93 ± 12.54 vs. 8.85 ± 10. 22; *p* ≤ 0.001) and mnestic domains (RAVLT: immediate recall 31.25 ± 7.31 vs. 36.28 ± 7.66; *p* = 0.001; RAVLT: delayed recall 5.60 ± 2.21 vs. 7.01 ± 2.10; *p* ≤ 0.001) at 6-month following the procedure. Additionally, ET patients show an improvement in memory domains (RAVLT: immediate recall 31.25 ± 7.31 vs. 36.73 ± 6.26; *p* ≤ 0.001; RAVLT: delayed recall 5.60 ± 2.21 vs. 7.02 ± 1.73; *p* ≤ 0.001) and in quality of life (QUEST 34.93 ± 12.54 vs. 9.77 ± 11.20; *p* ≤ 0.001) at 1-year following MRgFUS (Table 2).

**Table 2:** Change in neuropsychological and neurobehavioral scores between baseline, 6-month, and 1 year follow-up for PD and ET patients.

		PD patients				ET patients				
Neuropsychological and neurobehavioral tests	Baseline	6-month follow-up	1 year follow-up	*p* value 6 months	*p* value 1 year	Baseline	6-month follow-up	1 year follow-up	*p* value 6-month	*p* value 1 year
Mini Mental State Examination	26.64 ± 2.71	28.13 ± 1.99	28.09 ± 1.98	0.014	0.022	27.95 ± 1.96	28.42 ± 1.46	28.52 ± 1.42	0.186	0.063
Montreal Cognitive Assessment	22.64 ± 4.30	23.27 ± 4.05	23.41 ± 3.78	0.162	0.081	23.89 ± 5.39	24.18 ± 3.27	24.30 ± 3.53	0.777	0.737
Frontal Assessment Battery	14.18 ± 3.03	14.73 ± 2.62	14.82 ± 3.10	0.194	0.294	14.29 ± 3.10	15.64 ± 2.13	15.44 ± 2.44	0.004	0.023
Single letter-cued (phonemic) fluency test	28.56 ± 10.55	28.84 ± 13.30	30.10 ± 11.48	0.881	0.316	26.31 ± 9.17	27.91 ± 7.59	27.83 ± 7.87	0.199	0.164
Single letter-cued (semantic) fluency test	10.36 ± 2.89	10.41 ± 2.78	10.12 ± 3.16	0.919	0.592	10.46 ± 2.60	10.58 ± 2.82	10.76 ± 2.60	0.773	0.515
Rey Auditory Verbal Learning Test R.I	32.40 ± 8.07	34.52 ± 9.32	33.74 ± 9.08	0.185	0.356	31.25 ± 7.31	36.28 ± 7.66	36.73 ± 6.26	0.001	< 001
Rey Auditory Verbal Learning Test R.D	5.54 ± 3.38	7.05 ± 5.38	5.65 ± 3.04	0.150	0.962	5.60 ± 2.21	7.01 ± 2.10	7.02 ± 1.73	< 001	< 001
Raven’s Progressive Matrices	28.39 ± 4.84	28.00 ± 6.23	28.29 ± 6.33	0.278	0.808	28.87 ± 4.71	29.63 ± 4.05	29.35 ± 3.95	0.127	0.520
Hamilton Anxiety rating scale	6.14 ± 4.51	2.95 ± 3.12	2.55 ± 2.91	0.002	0.006	5.29 ± 5.44	2.50 ± 4.76	2.81 ± 4.39	0.001	0.015
Beck Depression Inventory-II	4.73 ± 3.30	2.86 ± 3.23	1.68 ± 2.46	0.081	0.003	2.96 ± 4.04	1.14 ± 1.96	1.89 ± 3.07	0.023	0.112
Quality of life in Essential Tremor Questionnaire	-	-	-	-	-	34.93 ± 12.54	8.85 ± 10.22	9.77 ± 11.20	< 001	< 001
Parkinson’s disease Questionnaire-8 (PDQ-8)	8.10 ± 2.97	3.11 ± 1.56	3.10 ± 2.34	< 001	< 001	-	-	-	-	-

When stratifying neuropsychological and neurobehavioral findings based on the treatment side, we observed distinct patterns of improvement depending on the targeted VIM (Table 3): when a left VIM thalamotomy was performed, a significant improvement was found in mnestic functions [(RAVL: immediate recall 31.26 ± 7.40 vs. 35.09 ± 8.63; *p* = 0.002; RAVLT: delayed recall 5.31 ± 2.58 vs. 6.98 ± 4.06; *p* ≤ 0.001; FAB 14.12 ± 3.01 vs. 15.21 ± 2.45; *p* = 0.003)], QUEST (36.27 ± 11.80 vs. 9.88 ± 9.99; *p* ≤ 0.001), PDQ-8 (7.47 ± 2.76 vs. 3.06 ± 1.53; *p* ≤ 0.001), HAM-A (6.02 ± 5.09 vs. 2.86 ± 4.36; *p* ≤ 0.001) at 6 months as well at 1 year [(RAVL: immediate recall 31.26 ± 7.40 vs. 34.89 ± 7.40; *p* = 0.003), BDI-II (3.84 ± 3.79 vs. 1.71 ± 2.73; *p* ≤ 0.001), HAM-A (6.02 ± 5.09 vs. 2.74 ± 3.91; *p* ≤ 0.001), QUEST (36.27 ± 11.80 vs. 9.48 ± 11.07; *p* ≤ 0.001), PDQ-8 (7.47 ± 2.76 vs. 3.11 ± 2.35; *p* ≤ 0.001)]. When a right VIM thalamotomy was performed, an improvement in the quality of life and in anxiety-depressive symptoms was observed, although it did not reach statistical significance (Table 3).

**Table 3:** Change in neuropsychological and neurobehavioral scores between baseline, 6-month, and 1 year follow-up finding by side (left/right).

		LEFT VIM thalamotomy				Right VIM thalamotomy				
Neuropsychological and neurobehavioral tests	Baseline	6-month follow-up	1 year follow-up	*p* value 6 months	*p* value 1 year	Baseline	6-month follow-up	1 year follow-up	*p* value 6 months	*p* value 1 year
Mini Mental State Examination	27.34 ± 2.42	28.45 ± 1.51	28.33 ± 1.68	0.004	0.010	27.54 ± 2.35	27.31 ± 2.51	28.31 ± 1.86	0.750	0.293
Montreal Cognitive Assessment	23.40 ± 4.99	23.77 ± 3.77	23.95 ± 3.41	0.080	0.008	23.00 ± 4.96	23.85 ± 5.27	23.57 ± 5.09	0.457	0.174
Frontal Assessment Battery	14.12 ± 3.02	15.21 ± 2.45	15.05 ± 2.87	0.003	0.032	15.00 ± 3.31	15.42 ± 1.98	15.85 ± 1,77	0.824	0.548
Single letter-cued (phonemic) fluency test	27.25 ± 9.93	28.24 ± 10.64	28.95 ± 10.00	0.381	0.077	27.61 ± 9.42	28.81 ± 9.36	28.24 ± 7.51	0.725	0.835
Single letter-cued (semantic) fluency test	10.41 ± 2.77	10.52 ± 2.92	10.46 ± 3.04	0.695	0.932	10.46 ± 2.47	10.39 ± 1.73	10.55 ± 1.43	0.957	0.939
Rey Auditory Verbal Learning Test R.I	31.26 ± 7.40	35.09 ± 8.63	34.89 ± 7.40	0.002	0.003	34.81 ± 8.68	38.04 ± 6.60	38.35 ± 9.50	0.189	0.241
Rey Auditory Verbal Learning Test R.D	5.31 ± 2.58	6.98 ± 4.05	6.24 ± 2.17	< 001	0.007	7.20 ± 3.42	7.32 ± 2.44	7.40 ± 3.96	0.688	0.813
Raven’s Progressive Matrices	28.69 ± 4.24	29.41 ± 3.98	29.10 ± 3.97	0.074	0.427	28.45 ± 7.44	25.81 ± 9.54	27.50 ± 10.01	0.059	0.611
Hamilton Anxiety rating scale	6.02 ± 5.09	2.86 ± 4.36	2.74 ± 3.91	< 001	< 001	3.42 ± 4.15	1.71 ± 1.49	2.42 ± 2.93	0.462	0.684
Beck Depression Inventory-II	3.84 ± 3.79	1.84 ± 2.81	1.71 ± 2.73	0.004	< 001	3.14 ± 4.10	2.28 ± 2.05	2.28 ± 2.30	0.833	0.684
Quality of life in Essential Tremor Questionnaire	36.27 ± 11.80	9.88 ± 9.99	9.48 ± 11.07	< 001	< 001	24.00 ± 10.00	1.66 ± 0.57	5.00 ± 4.58	0.250	0.250
Parkinson’s disease Questionnaire-8 (PDQ-8)	7.47 ± 2.76	3.06 ± 1.53	3.11 ± 2.35	< 001	< 001	9.50 ± 4.35	3.35 ± 1.70	3.00 ± 2.30	0.125	0.098

When assessing correlations between motor tests and neuropsychological or neurobehavioral tests at 1 year after treatment, a moderate negative correlation was found between the PDQ-8 score and the CRST total score (*r* = −0.467; *p* = 0.028), as well as between CRST total score and FAB score (*r* = −0.408; *p* = 0.004). A strong negative correlation was found between the FAB score and the MDS-UPDRS-III score at 1 year (*r* = −0.745; *p* ≤ 0.001).

## 5 Discussion

Our results support the long-term efficacy and cognitive safety of the MRgFUS treatment for ET and PD related tremor. Indeed, MRgFUS is recognized as an emerging procedure for treating tremor and other neurological disorders, gaining popularity in clinical and research settings worldwide (32). Its main advantage over other lesion techniques lies in its capability to promptly detect potential complications through real-time intraprocedural monitoring. This enables operators to address any adverse effects by adjusting the initial target position as needed. Persistent side effects and symptoms following the procedure, whenever present, typically remain mild and resolve within a few weeks due to the resorption of perilesional edema (33, 34). However, identifying potential cognitive disturbances following thalamotomy can be challenging since cognitive changes may emerge later and necessitate longitudinal evaluation for detection. The only way to exclude interference from the lesion with normal cognitive performances is to provide the patient with longitudinal follow-up evaluations at predetermined intervals. As previously discussed, although the VIM is mainly considered a motor relay station, it might secondarily contribute to cognitive functions since it is integrated into the indirect pathway connecting the prefrontal cortex and deep cerebellar nuclei (35, 36). Possible subtle cognitive complications after unilateral thalamotomy using different techniques have been described: unilateral gamma knife thalamotomy and radiofrequency thalamotomy may cause a decline in phonetic verbal fluency and deficits in visuospatial memory (6–9). On the other hand, MRgFUS has been associated with a higher cognitive safety profile as compared to other techniques (8, 10, 12–14, 37). As highlighted by a recent meta-analysis, preserved cognition following MRgFUS might be due to the generation of smaller, more precise lesions, due to real-time monitoring of the lesion and thermographic feedback (13). An intriguing comparison has also been made regarding the cognitive effects of thalamotomy vs. thalamic stimulation, leading to the conclusion that both techniques carry minimal overall risk of cognitive decline (7). Additionally, it was found that verbal fluency is more likely to decrease following both left-sided thalamotomy and thalamic stimulation (7). Results of the COGNIFUS part 1 study added further insight to the discussion by revealing the absence of cognitive dysfunctions at 6 months and showing an improvement in feelings of anxiety and quality of life in patients treated with MRgFUS (14).

The *COGNIFUS part 2 study* extended the cognitive follow-up to 1 year and showed an improvement in specific cognitive domains and skills including working memory, verbal memory, attention and cognitive flexibility. Specifically, from the comparison of the results obtained at 6 months and 1 year, some differences emerge. While at 6 months specific cognitive functions remained largely unchanged with an improvement in anxious symptoms, at 1 year a significant improvement was observed not only in anxiety and mood feelings but also in the memory domains and in frontal functions. In this regard, this study took a step forward in establishing nonmotor outcomes of unilateral MRgFUS thalamotomy, by supporting a potential role of the procedure in preventing the development of cognitive complications mediated by the establishment of maladaptive networks. The most immediate hypothesis to explain the improvement in cognitive performances in treated patients is that an improvement or cessation of tremor may result in greater well-being for the patients, with positive effects on their attentional state. Just recently, a study based on interpretative phenomenological analysis has explored the experiences of ET patients undergoing the treatment throughout the entire surgical process, from the days leading up to the procedure to those following it (33): after the procedure, all participants described the suppression of tremors as life-changing, with some expressing that it took them some time to psychologically adjust to what essentially became their new body (33). This demonstrates that tremor suppression has effects on the patient that go beyond the motor dimension and can significantly influence the psychological and cognitive spheres. An alternative hypothesis, which requires further confirmation from studies specifically designed for this purpose, is that thalamotomy may influence the functioning of subcortical networks that modulate the patient’s cognition, particularly in terms of cognitive flexibility and attentional tone. Our findings and previously available evidence do not support suggesting a reconfiguration of brain networks following thalamotomy. However, some clinical elements suggest further investigation in this direction. Indeed, it is known that the prefrontal cortex has wide projections to the mesolimbic, amygdala, and thalamic areas. Various studies investigated cortical activity changes associated with MRgFUS thalamotomy (38, 39). A recent study, based on the investigation of neural activity-related brain dynamic changes in regional cerebral blood flow through functional near-infrared spectroscopy (fNIRS), suggested that therapeutic MRgFUS can promote the remodeling of neuronal networks and changes in cortical activity in association with tremor improvement (38). Similarly, another study using fMRI demonstrated that MRgFUS thalamotomy not only suppress tremor symptoms but also rebalances atypical functional hierarchical architecture in ET patients (39). Specifically, MRgFUS VIM thalamotomy appears to perturb the global brain functional scaffold by influencing spatial information exchange and processing across modalities and areas (38). Other fMRI study in MRgFUS patients suggested that a temporary reconfiguration of the whole brain network occurs following the procedure, although the modalities of the subsequent reorganization are not still clearly understood (40, 41). Overall, this evidence indicates that the effect of VIM thalamotomy is not limited to the lesion in the target but also depends on the reorganization of extensive networks encompassing cerebello-striatal-thalamo-cortical circuits. However, a possible reorganization occurring after a temporary diaschisis remains only a hypothesis that should be investigated through further longitudinal network analysis studies. Another issue that deserves further discussion is the difference in cognitive changes observed in the two subgroups, patients with ET and PD, respectively. While in patients with PD the improvement primarily concerned quality of life and mood, a real enhancement in specific cognitive domains, particularly in memory, was confined to patients with ET. This likely reflects the underlying differences between the two disorders. Although sharing tremor, some cognitive dysfunction and personality changes, patients with ET and PD are profoundly different. Available scientific literature shows that patients with PD perform more poorly than ET patients in cognitive tasks such as attention, executive function, memory, and naming (42). Therefore, the effect of the procedure on cognitive functions may be more uncertain and weaker in patients with PD, requiring further evidence.

Strengths of our study include the prospective design, the longitudinal follow-up, the rigorous criteria adopted for the inclusion of patients and the use of a comprehensive neuropsychological battery for assessment. A limitation of the study is the potential occurrence of a learning effect when longitudinally assessing cognitive performances: however, setting the reassessment at 6 months and at 1 year appears to be the best compromise to ensure a sufficiently long follow-up without interference from potential learning effects or disease progression, the latter of which inherently carries the risk of independent cognitive decline. Although the utilization of alternate forms, which are accessible for most tests, may be proposed to mitigate any potential learning effect, it is primarily recommended for tests not encompassed in the current neuropsychological battery, such as the Paced Auditory Serial Addition Test (PASAT) and the Stroop Color and Word Test (SCWT) (43). Furthermore, subgroup analysis revealed that improvement in memory domains exclusively pertained to patients with ET. This allows us to exclude a learning effect at 6-month and 1 year, which would have been expected to manifest in both subgroups. In any case, caution is mandatory in interpreting the results, which need to be confirmed in studies with larger samples. Another limitation lies in the high drop-out rate among patients initially screened for inclusion in the study. This drop-out rate was mainly due to the geographic distance of patients, resulting in difficulty returning 1 year later for clinical follow-up. However, we must consider that this drop-out rate could also introduce a bias in our results. Some open questions remain and should be the focus of further investigations. For the majority of patients, the treated hemisphere was also the dominant one. When stratifying neuropsychological and neurobehavioral findings based on the treatment side, we observed significant changes only when a left VIM thalamotomy was performed. Interpretation of these results must be cautious because patients with right-sided lesions are much less represented in the included sample. Future studies with a larger sample size are needed to better examine the effect of the lesion side on cognitive performances and emotional state. Moreover, the recent authorization for staged bilateral MRgFUS thalamotomies further underscores the importance of longitudinal studies in assessing patients beyond their motor dimension: the ideal studies should combine clinical evaluation of patients, both in terms of motor and cognitive aspects, with analysis of functional changes within cortico-subcortical networks whose functioning appears to be influenced by VIM thalamotomy.

## Data availability statement

The data that support the findings of this study are available from the corresponding author GS, upon reasonable request.

## Author contributions

GS: Conceptualization, Formal analysis, Writing – original draft, Writing – review & editing, Data curation, Investigation, Methodology. PS: Conceptualization, Writing – original draft, Writing – review & editing, Investigation, Data curation, Formal analysis, Methodology. FB: Conceptualization, Writing – original draft, Writing – review & editing, Investigation. AC: Conceptualization, Writing – original draft, Writing – review & editing, Investigation. CMas: Conceptualization, Writing – original draft, Writing – review & editing, Investigation. MP: Conceptualization, Writing – original draft, Writing – review & editing, Investigation. AS: Conceptualization, Writing – original draft, Writing – review & editing, Investigation. AR: Conceptualization, Writing – original draft, Writing – review & editing, Investigation. EC: Conceptualization, Writing – original draft, Writing – review & editing, Investigation. CMar: Conceptualization, Writing – original draft, Writing – review & editing, Investigation. MM: Writing – original draft, Writing – review & editing, Conceptualization, Investigation. RT: Writing – original draft, Writing – review & editing, Conceptualization, Investigation. FP: Conceptualization, Investigation, Writing – original draft, Writing – review & editing, Data curation, Formal analysis, Methodology, Supervision.

## Supplementary material

SUPPLEMENTARY FIGURE 1 Statistically significant changes in neuropsychological and neurobehavioral scores following the procedure in PD patients. Asterisks indicate significant *p* value (^***^< 0.001, ^**^0.003).

SUPPLEMENTARY FIGURE 2 Statistically significant changes in neuropsychological and neurobehavioral scores following the procedure in ET patients. Asterisks indicate significant *p* value (^***^< 0.001, ^**^0.001).
