# Supplementary figures and images for: Cognitive safety of focused ultrasound thalamotomy for tremor: 1-year follow-up results of the COGNIFUS part 2 study
Source: Front Neurol. 2024 Jun 17;15:1395282. doi: 10.3389/fneur.2024.1395282 (PMC11215051; doi:10.3389/fneur.2024.1395282)

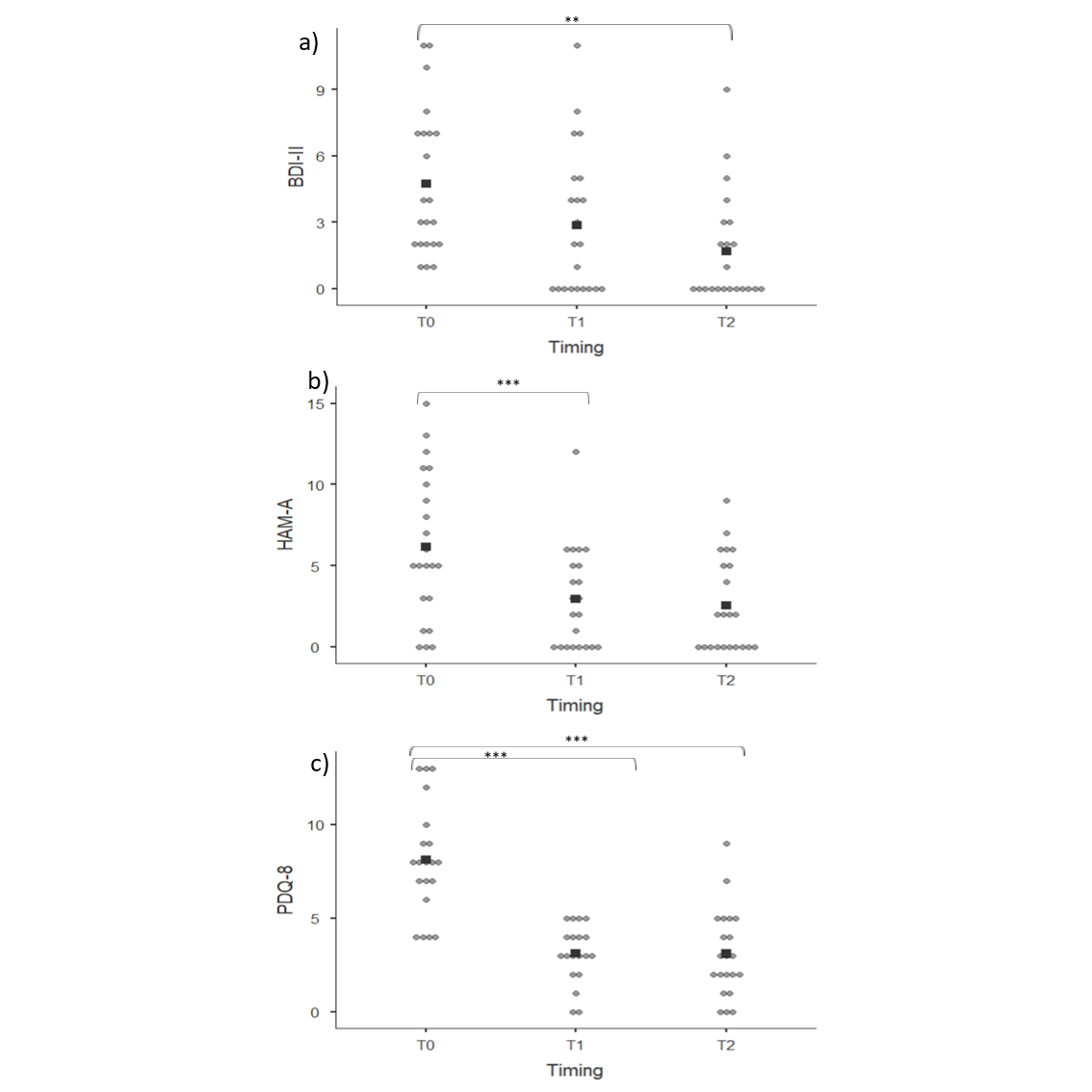

Supplement: SUPPLEMENTARY FIGURE 1 — Statistically significant changes in neuropsychological and neurobehavioral scores following the procedure in PD patients. Asterisks indicate significant p value (***< 0.001, **0.003). [file Image_1.TIF]

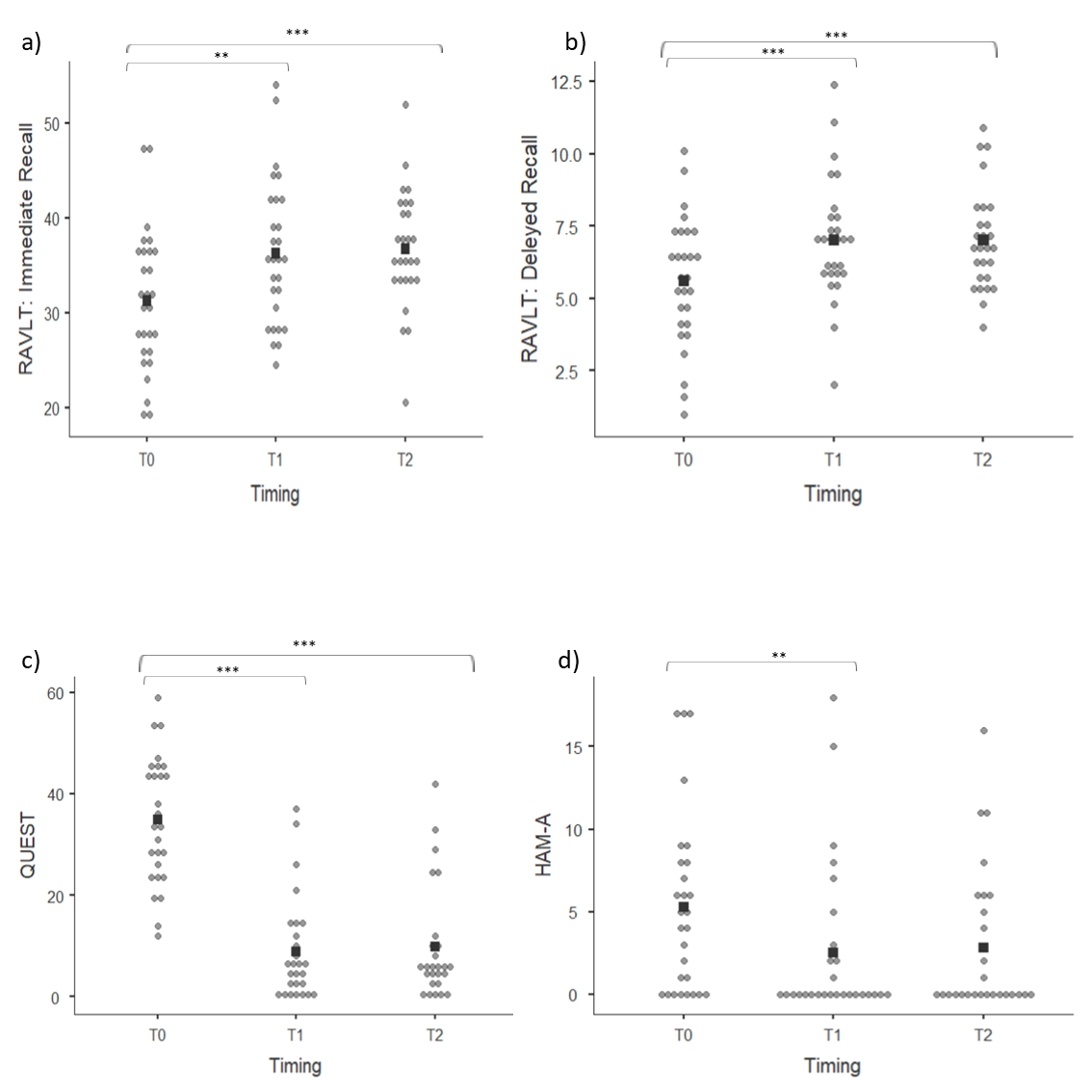

Supplement: SUPPLEMENTARY FIGURE 2 — Statistically significant changes in neuropsychological and neurobehavioral scores following the procedure in ET patients. Asterisks indicate significant p value (***< 0.001, **0.001). [file Image_2.TIF]
